# Necrotizing Enterocolitis: LPS/TLR4-Induced Crosstalk Between Canonical TGF-β/Wnt/β-Catenin Pathways and PPARγ

**DOI:** 10.3389/fped.2021.713344

**Published:** 2021-10-12

**Authors:** Alexia Gomart, Alexandre Vallée, Yves Lecarpentier

**Affiliations:** ^1^Département de Pédiatrie et Médecine de l'adolescent, Centre Hospitalier Intercommunal de Créteil, Créteil, France; ^2^Department of Clinical Research and Innovation, Foch Hospital, Suresnes, France; ^3^Centre de Recherche Clinique, Grand Hôpital de l'Est Francilien, Meaux, France

**Keywords:** necrotizing enterocolitis, NF-κB, transforming growth factor, PPARγ, canonical Wnt/β-catenin pathway

## Abstract

Necrotizing enterocolitis (NEC) represents one of the major causes of morbidity and mortality in premature infants. Several recent studies, however, have contributed to a better understanding of the pathophysiology of this dreadful disease. Numerous intracellular pathways play a key role in NEC, namely: bacterial lipopolysaccharide (LPS), LPS toll-like receptor 4 (TLR4), canonical Wnt/β-catenin signaling and PPARγ. In a large number of pathologies, canonical Wnt/β-catenin signaling and PPARγ operate in opposition to one another, so that when one of the two pathways is overexpressed the other is downregulated and *vice-versa*. In NEC, activation of TLR4 by LPS leads to downregulation of the canonical Wnt/β-catenin signaling and upregulation of PPARγ. This review aims to shed light on the complex intracellular mechanisms involved in this pathophysiological profile by examining additional pathways such as the GSK-3β, NF-κB, TGF-β/Smads, and PI3K-Akt pathways.

## Introduction

Necrotizing enterocolitis (NEC) is the most common and major cause of morbidity and mortality in premature infants (1–3). Morbidity is particularly significant in very premature babies treated in neonatal intensive care. The prevalence of NEC is about 7% in infants with a birth weight from 500 to 1,500 g (4). Mortality is 30% in pre-term infants born after 28 to 36 weeks of gestation and 40% after gestation of <28 weeks (5). Several risk factors have been reported in NEC, namely, prematurity, overfeeding, bacterial translocation, birth asphyxia, polycythemia, congenital heart disease, hyperosmolar formulas, maternal preeclampsia and respiratory distress syndrome. *Four* major pathways play a central role in the pathophysiology of NEC: bacterial lipopolysaccharide (LPS), toll-like receptor 4 (TLR4), canonical Wnt/β-catenin signaling and PPARγ. LPS activates TLR4 which in turn downregulates the Wnt/β-catenin pathway and upregulates PPARγ. Other pathways must also be discussed, i.e., NF-κB, TGF- β/Smads and PI3K-Akt pathways. Usually, canonical Wnt/β-catenin signaling and PPARγ are expressed in opposing ways so that when one pathway is upregulated, the other is downregulated and vice versa (6). It appears that in NEC, the canonical Wnt/β-catenin pathway is downregulated and PPARγ is up regulated through the complex inhibiting effects of TLR4, itself activated by LPS. This review discusses this profile, with a particular focus on the interactional roles of the NF-κB, TGF-β/Smads and PI3κ-Akt pathways. Moreover, in premature infants, a genetic predisposition to NEC has been reported (7–9). Inherited defects in the regulation of innate immune pathway contribute to NEC susceptibility in premature newborns.

## Histology and Pathophysiology of NEC

The intestinal epithelium represents the first barrier of defense against luminal agents. It remains in permanent turnover and is progressively replaced by intestinal stem cells (ISCs). These cells are localized within the intestinal crypts and maintain the integrity and viability of the epithelium, necessary for gut regeneration. During NEC, loss of ISCs leads to severe gut damages (10). In premature infants, the intestinal mucosa can be altered by various factors such as hypoxia, infections and administration of non-maternal milk (11–14). Main histological findings in NEC consist of bacterial overgrowth, inflammation and ischemic necrosis of the intestine. In premature infants, NEC is associated with an immune response to the gut microbiota in the intestinal tract, leading to inflammation and lesions that can result in infarction (15). Intestinal damages range from alteration of the intestinal mucosa to perforation and necrosis. The terminal ileum and the large intestine are usually affected and, in the most severe cases, the entire intestinal tract is subject to thickening of the intestinal wall, edema of the intestinal mucosa, areas of fibrinous adhesions, bleeding sites, areas of intestinal stenosis and bands of transmural necrosis secondary to bacterial infiltration and gas collections. The initial lesion in the small intestine consists of a loss of intestinal villi by apoptosis, leading to local weakening of the intestinal barrier and to translocation of bacteria and other elements present in the intestinal lumen, responsible for intestinal inflammation (16, 17). In response to the loss of continuity in the intestinal epithelium, a repair-healing program begins. This involves the migration of healthy enterocytes at the level of the injured parts, in order to seal the intestinal mucosa and to limit bacterial translocation (18). The generation of new enterocytes takes place in the crypts of Luberkuhn (19–22). In premature infants, enterocyte migration and proliferation are largely inhibited, which limits the intestinal repair processes (23).

## Dysmaturity of Intestinal Immune Response in NEC (24, 25)

NEC is rare in full term neonates and can appear in a context of ischemia (26). This suggests a dysmature intestinal immune response in NEC. In mouse fetus, the TLR4 signaling plays a central role in crypt development and intestinal epithelial cell (IEC) differentiation (27). After preterm birth, a persistent activation of the IEC TLR4 pathway is potentially pathological, due to the fact that gut bacteria can induce intestinal inflammation and necrosis via TLR4 activation. In term neonates, there is a rapid desensitization of the IEC TLR pathway and acquisition of a postnatal intestinal tolerance (28). This is a consequence of a decreased expression of IRAK1, which is a mediator of the TLR pathway, and inhibition of NFκB (28–30). In IEC, TLRs maintain both the mucosal homeostasis and the IEC function while contributing to inflammation and necrosis. Thus, in the preterm intestine, there is an imbalance between pro-inflammatory and anti-inflammatory immune processes, and the expression of inhibitors of the TLR pathway, such as SIGIRR, A20, and TOLLIP, is decreased (31). In the mouse intestine, breast milk and probiotics prevent NEC and upregulate the expression of genes that inhibit the TLR4 pathway (32–34).

## Lipopolysaccharide (LPS) and Toll-Like Receptor 4 (TLR4)

### Bacterial Lipopolysaccharide (LPS)

Lipopolysaccharides (LPS) are endotoxins found in the outer membrane of Gram-negative bacteria. They consist of three parts: O antigen, core oligosaccharide and lipid A, which is considered to be toxic on epithelial cells and granulocytes. LPS induces a strong response from the immune system. LPS binds with the TLR4 receptor and promotes the release of several pro-inflammatory cytokines. LPS plays a key role in NEC and patients with NEC present high levels of plasmatic LPS. Intraperitoneal injection of LPS in mice and rats induces intestinal injury. LPS is the ligand for TLR4 that mediates NEC (23, 35, 36).

### Bacterial LPS Toll-Like Receptor 4 (TLR4)

TLR4 is a transmembrane protein and member of the toll-like receptor family, which belongs to the pattern recognition receptor (PRR) family. Activated TLR4 leads to activation of the NF-κB pathway and production of numerous inflammatory cytokines. TLR4 is an important mediator of the innate immunity. TLR4 drives the inflammatory response by activation of pro-inflammatory agents (37). TLR4 plays a key role in inflammatory processes such as sepsis (38), ulcerative colitis (39–41) and atherosclerosis (42). It also plays a central role in NEC pathogenesis (23, 35, 43, 44). TLR4 expression and function are upregulated in premature infants. Indeed, inside the intestinal epithelium of premature infants, activation of TLR4 by LPS alters the intestinal mucosa and reduces epithelial repair mechanisms. Moreover, TLR4 is a mediator of innate immunity in macrophages and is linked to a susceptibility to inflammatory bowel diseases (45–48).

### TLR4 Activation on the Intestinal Epithelium in NEC

#### LPS-Induced TLR4 Activation Inhibits Enterocyte Migration in the Intestinal Epithelium in NEC

TLR4 is expressed in the intestinal epithelium. NEC is characterized by a marked defect in the migration of enterocytes, leading to serious alterations of the intestinal mucosa (23, 49–51). Activation of TLR4 diminishes the migration of enterocytes via an increase in actin stress fibers. This increases the adhesion of enterocytes to the basal membrane (52). TLR4 increases the activity of the GTP-ase RhoA which itself activates the formation of actin stress fibers via activation of the focal adhesion kinase (FAK). FAK enhances adhesion of enterocytes to the intestine wall (23, 49). TLR4 also displaces β-1 integrins and this further restricts cell movements. Thus, β-1 integrin antibodies or FAK inhibitors reverse the deleterious effects of TLR4 activation on enterocyte migration. This is mediated by the release of pro-inflammatory cytokines such as interferon (53), which in turn inhibits enterocyte migration via the Connexin 43 (54). Nitrite oxide inhibits enterocyte movements via the activation of RhoA (50).

#### LPS-Induced TLR4 Activation on the Intestinal Epithelium Inhibits Enterocyte Proliferation in NEC

The loss of enterocytes initiates the migration of enterocytes from healthy to diseased areas. This induces the proliferation of enterocytes from stem cells located in the intestinal crypts. However, this proliferation is reduced during NEC (1, 55). NEC development requires activation of the innate immune TLR4 in enterocytes. TLR4 activation by LPS inhibits enterocyte proliferation and favors NEC progression (49, 56). Activation of TLR4 alters enterocyte proliferation in the ileum of newborn mice and in cultured enterocytes (1). Enterocyte proliferation is also inhibited after endotoxin exposure (57, 58).

#### LPS-Induced TLR4 Activation on Intestinal Stem Cells Leads to Their Loss Through Apoptosis in NEC

During intestinal aggression, the regular and rapid turnover primarily involves stem cells or progenitors located at the base of the intestinal crypts (59), Bmi1 (60–62), and Lgr5 (63–67). TLR4 is expressed in Lgr5-positive intestinal stem cells (23). TLR4 activation increases apoptosis through upregulation of p53, a modulator of apoptosis (PUMA). Inhibition of PUMA *in vivo* restores proliferation and reduces apoptosis in NEC.

In enterocytes, TLR4 promotes enterocyte apoptosis. In experimental NEC, a significant decrease in the number of enterocytes is observed at an early stage due to an increase in apoptosis processes (17, 68–73). This favors the transmural passage of infectious agents and the activation of immune processes. Within the intestinal epithelium, early activation of TLR4 promotes the loss of enterocytes (1, 23, 27, 74). The TLR4 deficient mouse is protected from the development of NEC. Moreover, inhibition of TLR4 in the epithelium of newborn mice prevents the development of NEC and decreases enterocyte apoptosis (1, 23, 74). Inhibition of TLR4 activation within the intestinal epithelium enhances enterocyte proliferation (1, 75) and inhibits enterocyte apoptosis in the small intestine of the premature host (10, 18).

In human infant NEC, there is an increased expression of TLR4 while inhibitors of TLR signaling, such as SIGIRR, are decreased (32–34, 76). In preterm infants with NEC, microbiota presents numerous Gram-negative bacteria suggesting that TLR activation plays a role in human NEC pathogenesis (77, 78). In NEC C3HeJ mice that lack the functional TLR4 signaling (35), IEC-specific deletion of TLR4 induces protection against experimental NEC. Mice lacking SIGIRR, a negative regulator of the TLR pathway, present severe experimental NEC (7, 79).

## Generalities On Canonical WNTβ-Catenin Signaling and PPARγ

### Canonical Wnt/β-Catenin Signaling

**Figure 1:**
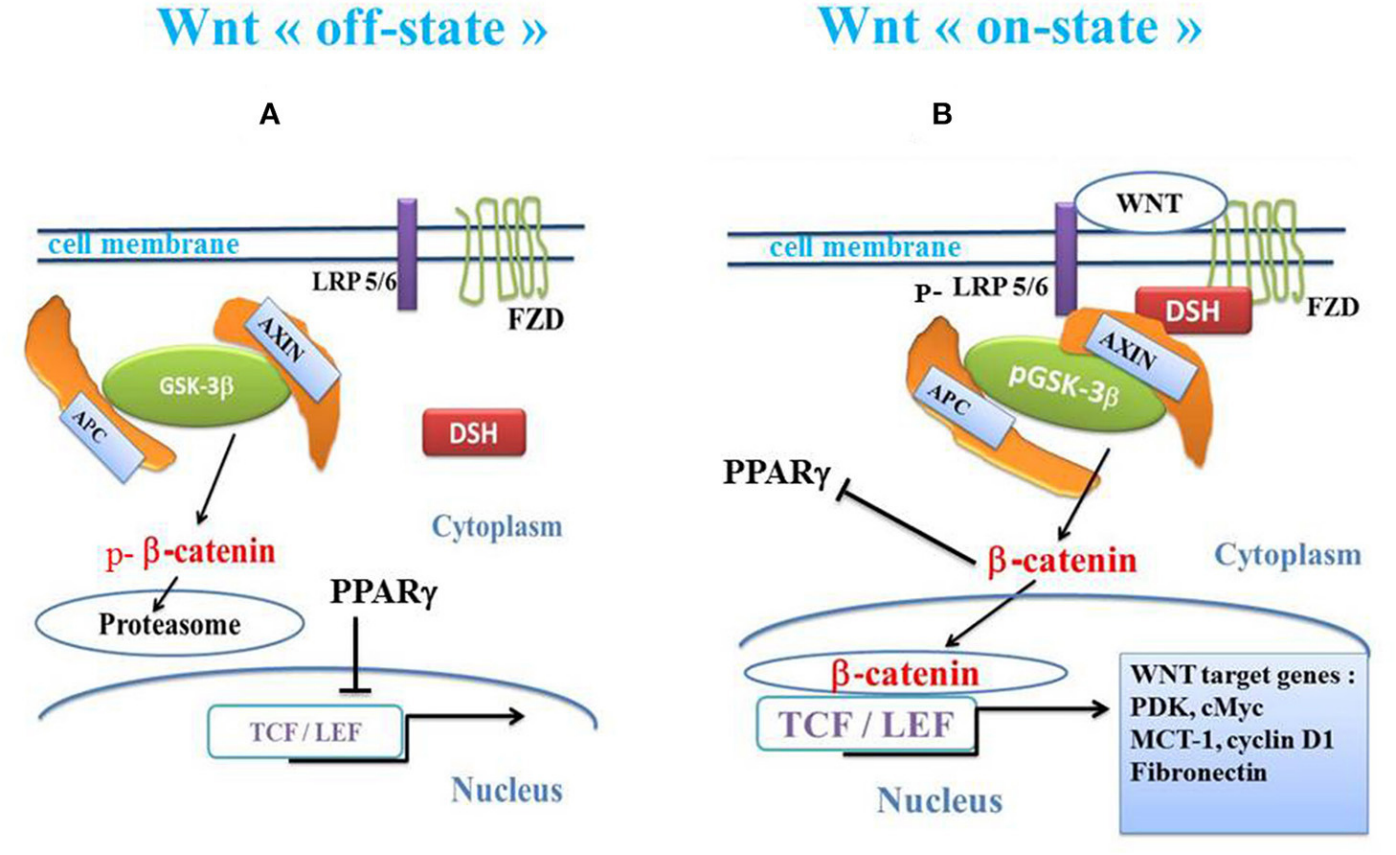
Schema of canonical Wnt/β-catenin pathway and PPARγ. **(A)** In the absence of the WNT ligands (“off state”), cytosolic β-catenin is phosphorylated by GSK-3β. APC and AXIN combine with GSK-3β. Thus, p-β-catenin induces the destruction process and migrates to the proteasome. PPARγ inhibits the β-catenin /TCF-LEF-induced activation of the Wnt target genes. **(B)** In the presence of the Wnt ligands (“on state”), a Wnt agent binds both Frizzled and LRP5/6 receptors to initiate LRP phosphorylation and disheveled-mediated Frizzled internalization. GSK-3β is phosphorylated and β-catenin phosphorylation is inhibited, which prevents its degradation into the proteasome. Thus, β-catenin accumulates in the cytosol and translocates to the nucleus to bind TCF-LEF co-transcription factors. This induces the Wnt-response gene transcription (PDK, MCT-1, Myc, Cyclin D1, fibronectin). β-catenin inhibits PPARγ. APC, adenomatous polyposis coli; DSH, Disheveled; FZD, Frizzled; GSK-3β, glycogen synthase kinase-3β; LRP5/6, low-density lipoprotein receptor-related protein 5/6; PPARγ, peroxisome proliferator-activated receptor gamma; TCF/LEF, T-cell factor/lymphoid enhancer factor; MCT-1, monocarboxylate lactate transporter-1.

The canonical Wnt/β-catenin pathway plays a key role in cell fate, metabolism, epithelial-mesenchymal transition (EMT) and embryogenesis (80–82). In the presence of Wnt ligands, the canonical Wnt receptor is linked with both Frizzled (FZD) and the LDL receptor-related protein 5/6 (LRP5/6). FZD, which is linked to Disheveled (DSH), disrupts the destruction complex composed of the tumor suppressor adenomatous polyposis coli (APC), AXIN and glycogen synthase kinase-3β (GSK-3β). GSK-3β negatively regulates β-catenin by phosphorylation leading to its degradation into the proteasome. PI3K-Akt signaling is a negative regulator of GSK-3β, inactivating GSK-3β by phosphorylation of its Ser 9 residue leading to pGSK-3β (83). The activated receptor finally results in the inhibition of the β-catenin phosphorylation, thereby preventing degradation of the β-catenin by the proteasome. The stabilized β-catenin then accumulates in the cytoplasm, translocates to the nucleus and interacts with the transcription factor T-cell /lymphoid enhancer (TCF/LEF) to activate numerous β-catenin target genes such as cyclin D1, MMP7, c-Myc, and fibronectin (84–86). In the absence of Wnt ligands, β-catenin is phosphorylated by the destruction complex and then degraded in the proteasome (Figure 1).

In enterocytes, LPS activates GSK-3β and increases the phosphorylation of β-catenin, leading to its proteasomal degradation. Phosphorylation of GSK-3β is mediated by the phosphorylation of the PI3K-Akt pathway (87). LPS causes a decrease in PI3K-Akt phosphorylation. siRNA knockdown of GSK-3β completely reverses the inhibitory effects of LPS on enterocyte proliferation. Lithium chloride reverses the LPS-induced inhibition of the nuclear translocation of β-catenin in IEC-6 cells (1). TLR4 and the PI3K-Akt/GSK-3β signaling mediates the inhibitory effects of LPS on enterocyte proliferation (1). NEC is associated with TLR4-mediated inhibition of the pGSK-3β/β-catenin pathway in enterocytes. Inhibition of enterocyte proliferation in mice is similar to that occurring in human infants with NEC.

### PPARγ

PPARγ is a transcriptional factor belonging to the nuclear hormone receptor superfamily. It heterodimerizes with the retinoid X receptor. PPARγ is expressed in numerous cell types, such as adipose tissues, muscles, brain, and immune cells. It regulates innate immune responses, insulin sensitivity, inflammation, glucose and lipid homeostasis, and cell fate (88, 89). PPARγ is activated by synthetic ligands such as PPARγ agonists thiazolidinediones (TZDs) and natural agents such as 15d-prostaglandin J2. TZDs improve insulin sensitivity in peripheral tissues and ameliorate glucose tolerance and insulin sensitivity in type 2 diabetic patients. Abnormalities of PPARγ are observed in several pathological states such as cancers, diabetes, obesity, and atherosclerosis. Some TZDs have been used for treating type 2 diabetes. PPARγ also plays an important role in regulating cardiovascular rhythms by controlling circadian variations of blood pressure and heart rate through BMAL1 (6, 90, 91).

### Crosstalk Between the Canonical Wnt/β-Catenin and PPARγ

In numerous diseases, the canonical Wnt pathway is generally regulated in an opposing manner to that of PPARγ (6). If one of them is downregulated, the other is upregulated and vice versa. Numerous studies have shown the direct interaction between β-catenin and PPARγ (92–96). PPARγ activation inhibits the β-catenin activity of TCF/LEF transcription factors (97). TZDs-PPARγ agonists (troglitazone, rosiglitazone and pioglitazone) and the non-TZD PPARγ activator GW1929 inhibit the β-catenin-induced transcription in a PPARγ-dependent manner. PPARγ ligands repress the canonical Wnt pathway via the PI3K/Akt signaling (98).

Some diseases are characterized by downregulation of the canonical Wnt/β-catenin signaling and upregulation of PPARγ. Conversely, numerous diseases are characterized by upregulation of the canonical Wnt/β-catenin signaling and downregulation of PPARγ (6, 90, 91, 99).

## Transforming Growth Factor-β (TGF-β) and SMADS

**Figure 2:**
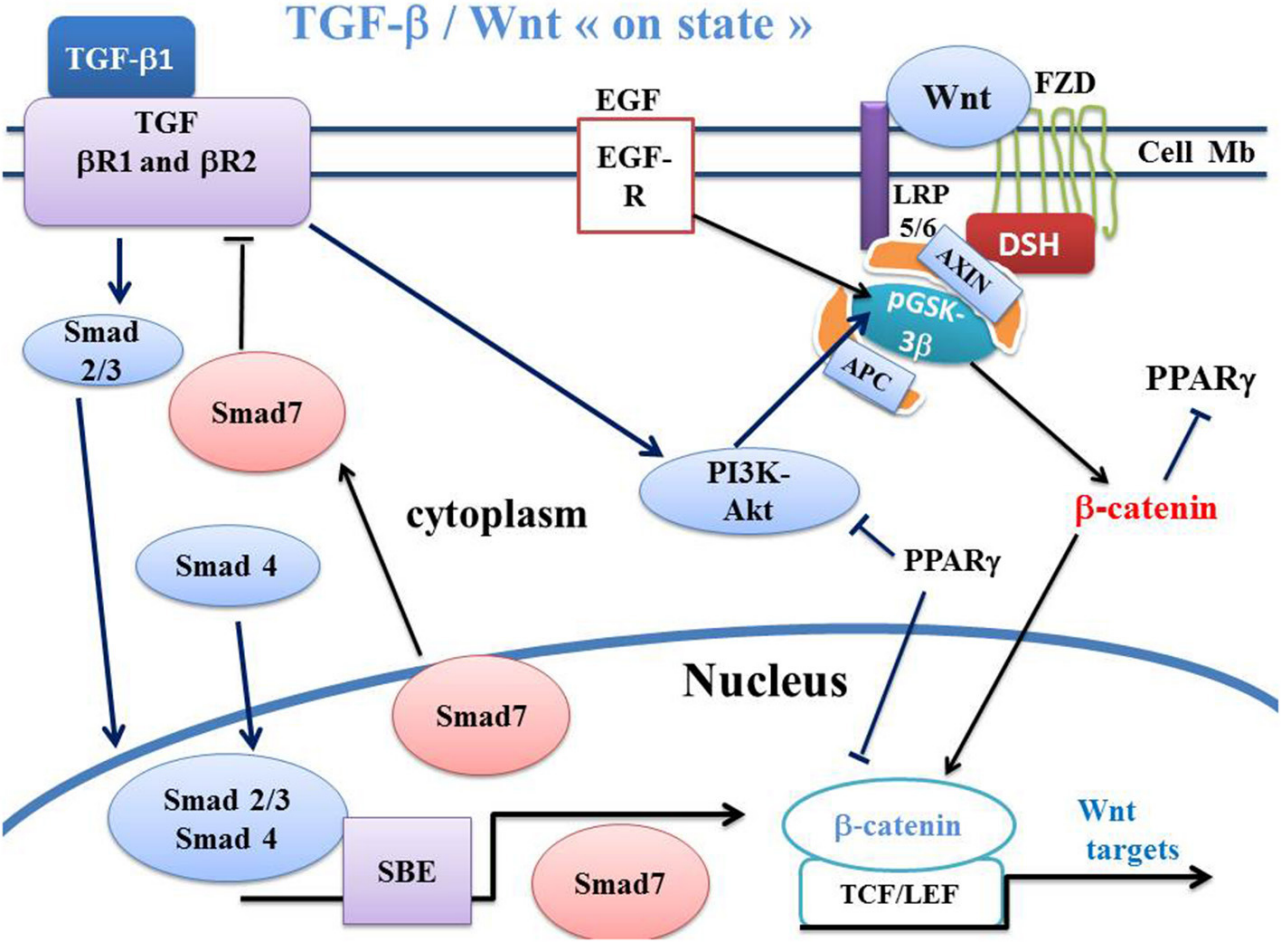
Schema of canonical Wnt/β-catenin, TGF-β, SMAD pathways and PPARγ in Wnt “on state.” In canonical Wnt signaling “on state,” TGF-β1 binds the type 2 TGF-βR2 receptor (TGF-βR2), Thus, TGF-βR2 recruits the type 1 TGF-βR1 receptor (TGF-βR1). This induces the formation of a heterotetramer that phosphorylates Smad. The Smad complex then translocates to the nucleus and regulates the transcription of Smad target genes. Smad7 in turn inhibits the TGF-βR1-R2 receptors. The PI3K-Akt pathway is a non-Smad signaling which is activated by TGF signaling and which induces phosphorylation of GSK-3β. PPARγ inhibits PI3KAkt and the β-catenin/TCF-LEF-induced activation of WNT target genes. APC, adenomatous polyposis coli; DSH, Disheveled; FZD, Frizzled; GSK-3β, glycogen synthase kinase-3β; LRP5/6, low-density lipoprotein receptor-related protein 5/6; PPARγ, peroxisome proliferator-activated receptor gamma; PI3K, phosphatidylinositol 3-kinase; Akt, Akt/Protein Kinase B; TCF/LEF, T-cell factor/lymphoid enhancer factor; TGF, Transforming Growth Factor; SBE, Smad binding element; EGFR, Epidermal growth factor receptor.

**Figure 3:**
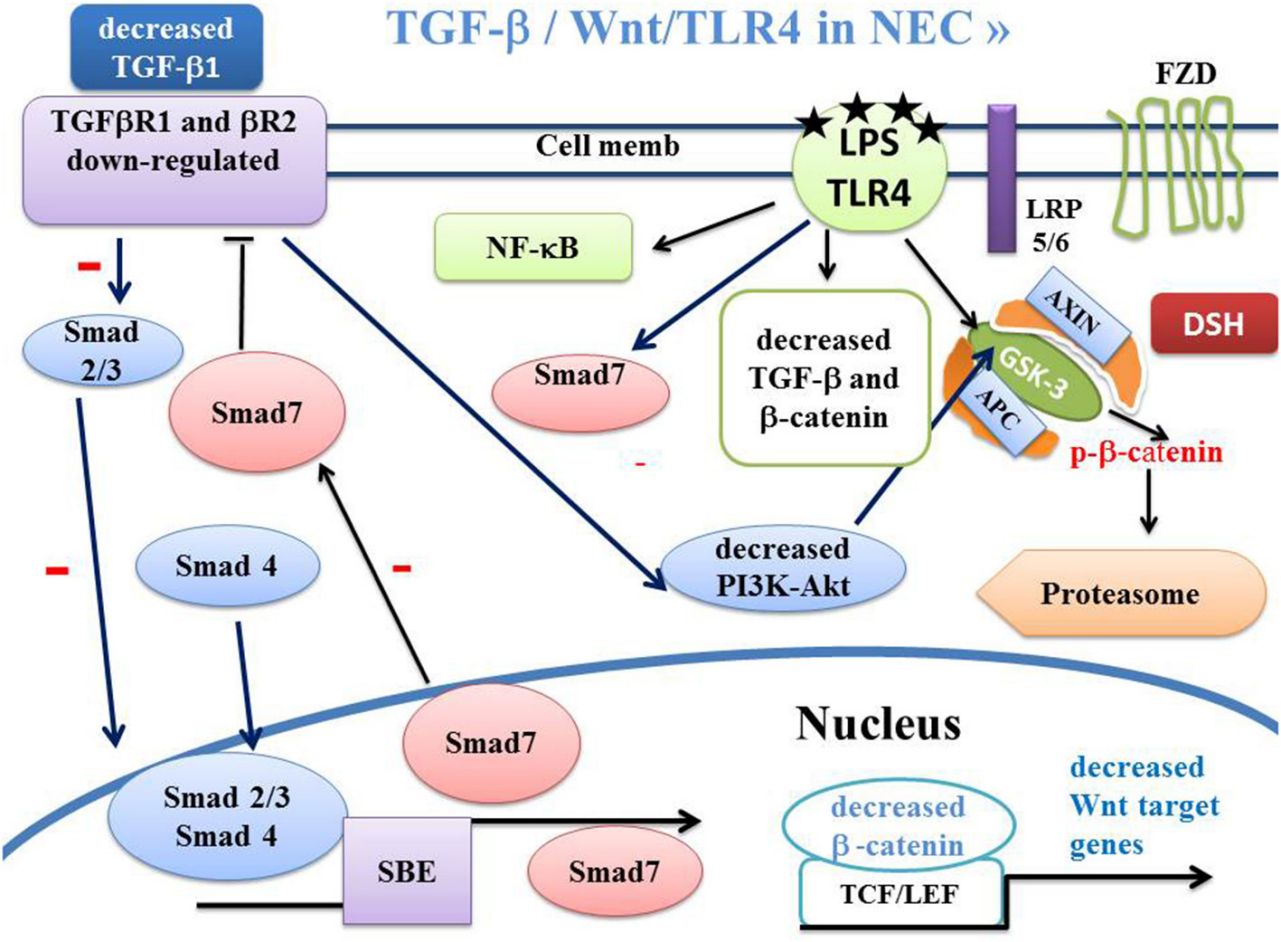
Schema of canonical Wnt/β-catenin, TGF-β, SMAD and PPARγ pathways in NEC. In NEC, the LPS-TLR4 complex induces inactivation of TGF-β signaling. This leads to dephosphorylation of GSK-3β, inducing β-catenin phosphorylation and β-catenin translocation into the proteasome. The LPS-TLR4 complex also inactivates the PI3K-Akt pathway leading to dephosphorylation of GSK-3β and β-catenin translocation to the proteasome. APC, adenomatous polyposis coli; DSH, Disheveled; FZD, Frizzled; GSK-3β, glycogen synthase kinase-3β; LRP5/6, low-density lipoprotein receptor-related protein 5/6; PPARγ, peroxisome proliferator-activated receptor gamma; PI3K, phosphatidylinositol 3-kinase; Akt, Akt/Protein Kinase B; TCF/LEF, T-cell factor/lymphoid enhancer factor; TGF, Transforming Growth Factor; SBE, Smad binding element.

TGF-β is a growth factor involved in numerous physiological processes such as embryonic development, tissue repair, differentiation and cell growth (100). Three TGF-β isoforms, TGF-β1, TGF-β2, and TGF-β3, are encoded by three distinct genes. After activation, TGF-β induces a cellular response by binding to specific type I and type II receptors (TβRI and TβRII, respectively). TGF-β binds with TβRII. Signal transduction from receptors to the nucleus is provided primarily by the phosphorylation of Smad proteins (101). The Smad family is divided into three different functional groups: the Smads associated with the receptors or R-Smads, which specifically interact directly with the activated TβRI; the co-Smads, common mediators for all members of the TGF-β family; and the inhibitory Smads or I-Smad. TGF-β1 binds with TGF-βR2 which recruits TGF-βR1 This forms a heterotetramer that phosphorylates Smad2 and Smad3 which bind with Smad4 (Figures 2, 3). This complex translocates to the nucleus where it binds with the Smad binding element DNA sequences, and acts as a transcription factor. Smad7, an I-Smad, binds with the activated TβRI, thus preventing phosphorylation of Smad2 and Smad3. Smad7 blocks TGF*-*β signaling by preventing the complex formation between Smad2 or Smad3 and Smad4 and the nuclear accumulation of Smad2 and Smad3 in response to TGF*-*β signaling (102). On the other hand, Smad7 is a general antagonist of the TGF*-*β family through the TGF*-*β type 1 receptor (103). The inhibitory action of Smad7 functions as a negative feedback loop. Smad7 acts by blocking the TGF-β-induced growth inhibition and apoptosis. There is a high Smad7 expression both in the uninflamed preterm intestine and during NEC, where it blocks the normal autocrine induction of TGF-β2 in epithelial cells. Smad7 can suppress TGF-β signaling by competing with activating Smads, interfering with the TGF-β receptor function and increasing its degradation. Smad7 promotes the inflammatory activation of NEC macrophages and interrupts the TGF-β signaling in intestinal macrophages during NEC (104). TGF-β1 downregulates PPARγ expression in various systems via the Smad pathway (105–107). TGF-β1 activates the canonical Wnt signaling. The link between TGF- β1, canonical WNT/β-catenin and PPARγ has been well-documented (95, 96, 108). Thus, TGF-β1 stimulates canonical WNT signaling and represses PPARγ. In NEC, interruption of TGF-β signaling partly explains the upregulation of PPARγ, given that these two pathways act in an opposing manner (Figure 3).

## Nuclear Factor Kappa-Light-Chain-Enhancer of Activated B Cells (NF-κB)

The transcription factor NF-κB consists of five subunits (p50, p65, p52, cRel, and RelB). These subunits heterodimerize to form the active factor NF-κB (36). p50-p50 and p50-p65 are the NFκB dimers mostly found in intestinal tissues (109, 110). NF-κB is constitutively present in the cytoplasm of most cells, in an inactive state, as it is bound to the inhibitory proteins IκB. NF-κB binds with the inhibitor IκB kinase (IKK) in the cytoplasm and exists in an inactivated form. Phosphorylation of IκB dissociates it from NF-κB. Activation of NF-κB signaling is initiated by the degradation of the IκB proteins via activation of the IκB kinase. IKK is composed of a heterodimer of the IKKα and IKKβ subunits and of the regulatory protein NEMO which catalyzes the phosphorylation of the IkB proteins. IkBs are destroyed by the proteasome, allowing NF-kB (RelA–p50 heterodimer) to translocate to the nucleus where it induces the expression of specific genes. NF-κB controls DNA transcription, cytokine production and cell survival. Activation of NF-κB upregulates the production of inflammation-related proteins. NF-κB is a rapid-acting primary transcription factor, and is present in cells in an inactive form that does not require a protein synthesis to become active. NF-κB is involved in cellular responses to stimuli such as stress, cytokines, ROSs, tumor necrosis factor alpha, interleukin 1-β, and the bacterial LPS-TLR4 pathway (111). The canonical Wnt/NF-κB signaling appears to be complex (112, 113). Many bacterial products lead to NF-κB activation and induce rapid changes in gene expression (114). NF-κB signaling plays an important role in NEC. Activation of NF-κB in the intestine appears at 20 days of gestation in fetal rats, i. e., at the end of the gestation period of 21 days (115). LPS is a potent activator of the transcription of NF-κB (36, 116).

## PI3K-AKT/GSK-3β Signaling

The TLR4 pathway blocks PI3K-Akt phosphorylation, allowing GSK-3β to remain active (i.e., under the GSK-3β unphosphorylated form) and to phosphorylate β-catenin leading to its destruction into the proteasome (Figure 1). Inhibition of GSK-3β with lithium chloride, which phosphorylates GSK-3β, prevents the negative effects of LPS on β-catenin and restores proliferation of the IEC-6 cells (117). In mice IECs and after dextran sodium sulfate (DSS)-induced injury, activation of TLR4 signaling increases the expression of cyclo-oxygenase (COX)-2, prostaglandin E2 (PGE2), and endothelial growth factor receptor ligands. This leads to a decrease in proliferation (118). In NEC, both COX-2 and PGE2 play a key role in gut homeostasis and inflammation (119, 120). The beneficial effect mediated by stimulation of the canonical Wnt pathway may act through inhibition of COX-2/PGE2 signaling. Formula-feeding in mice induces an increase in the non-phosphorylated GSK-3β protein, leading to a decrease in β-catenin in the ileum in NEC newborns.

## Crosstalk Between TLR4 Signaling and the Canonical WNT/β-Catenin Pathway

### Activation of TLR4 Inhibits the Canonical Wnt/β-Catenin Pathway (Excluding NEC)

The first link between TLR4 and β-catenin was established by Ireland et al. (121). In a transgenic line (Ahcre) of adult mice, β-catenin has been shown to be required for the maintenance of a small intestinal cell proliferation and is implicated in goblet cell differentiation. Excessive activation by LPS-TLR4 /NF-κB signaling inhibits the canonical Wnt/β-catenin pathway and induces steroid-associated necrosis of femoral head (SANFH) in rats (122). TLR4 inhibits the canonical Wnt/β-catenin pathway, decreases activation of the Wnt receptor LRP6, and blocks the protective effect of the Wnt3a ligand (123). The knockout of TLR4 activates canonical Wnt/β-catenin signaling and promotes fracture healing (124). In skin wounds, activated Wnt7b favors regeneration via PGE2 which is a by-product of COX-2 (125).

### Activation of TLR4 Inhibits the Canonical Wnt/β-Catenin Pathway in NEC

Several studies show that canonical Wnt/β-catenin signaling is downregulated in NEC. TLR4 activation leads to decreased β-catenin and increased GSK-3β expression in the intestinal mucosa in both human and murine NEC (1, 24, 126–128). On the other hand, in enterocytes *in vivo*, inhibition of TLR4 signaling reverses these effects and restores levels of enterocyte proliferation in experimental NEC. TLR4 inhibits enterocyte proliferation via the inhibition of β-catenin signaling. LPS causes a dose-dependent decrease in β-catenin expression in the nucleus. This occurs via an increase in the dephosphorylated form of GSK-3β, which is restricted to the small intestine of newborn mice (1).

In both mice with experimental NEC and infants with acute active NEC, the canonical Wnt/β-catenin signaling and intestinal regeneration are decreased. LPS-induced activation of TLR4 leads to dephosphorylation of pGSK-3β (1). An increase in the GSK-3β form leads to the degradation of the β-catenin into the proteasome. Importantly in mice with NEC, exogenous Wnt7b repairs intestinal injury and restores the ISC function and the intestinal epithelial homeostasis (24) (Wnt7b is a ligand for members of the frizzled family of receptors that is involved in canonical Wnt/β-catenin signaling). Moreover, a Wnt7b supplementation attenuates intestinal injury by rescuing ISCs and restoring intestinal regeneration (129). The Wnt/β-catenin pathway controls the proliferation and maintenance of ISCs (130). The canonical Wnt signaling regulates ISCs and promotes the formation of Paneth cells (131). Wnt ligands are released from the Paneth cells (132). β-catenin is essential for intestinal homeostasis and maintenance of ISCs and promotes intestinal cell proliferation (133).

In both mice and humans, NEC is associated with decreased β-catenin signaling (24). In NEC intestinal mucosa, inhibition of the enterocyte β-catenin pathway can be reversed, and enterocyte proliferation is restored through an adenoviral-mediated inhibition of TLR4 signaling. In NEC mice, inhibition of TLR4 in enterocyte signaling restores enterocyte proliferation, reverses inhibition of β-catenin expression and favors GSK-3β phosphorylation (1). Animals with mutant or deficient TLR4 pathways are protected against NEC (23, 35). From a preventive and therapeutic viewpoint, this highlights the key role of the canonical Wnt/β-catenin pathway in NEC (1, 134).

### Downregulation of TGF-β Signaling in NEC

The link between TGF-β1, canonical Wnt/β-catenin and PPARγ has been well-documented (96, 106, 108). TGF-β1 has been shown to activate canonical Wnt signaling and to inhibit PPARγ. TGF-β1 activates the Smad pathway and non-Smad pathways as MAPK and PI3K-AKT. TGF-β1 downregulates PPARγ expression in various systems via the Smad pathway (105–107). TGF-β signaling is downregulated in NEC. A low blood TGF-β1 level is associated with NEC in infants with an extremely low birth weight (ELBW) (135). Blood TGF-β1 is a biomarker used to estimate the risk of NEC in a newly-born premature infant.

NEC is associated with a decreased intestinal tissue expression of TGF-β. In an LPS mouse model, the disruption of the TGF-β pathway results in a severe NEC-like mucosal injury (136). Macrophage cytokine production is decreased in the developing intestine by TGF-β, particularly the TGF-β2 isoform (136). The TGF-β2 isoform suppresses macrophage inflammatory responses in the developing intestine and protects against inflammatory mucosal injury. Smad7 is an important negative regulator of TGF-β signaling in the gastrointestinal tract (137). Smad7 inhibits TGF-β signaling by competing with activating Smads, thus interfering with the TGF-β receptors and increasing their degradation. Bacterial products induce Smad7 expression in neonatal macrophages.

Smad7 upregulates IKK-β expression in macrophages through direct binding and transcriptional activation of the IKK-β promoter. Smad7 interrupts the TGF-β signaling in intestinal macrophages and promotes inflammatory activation of these cells during NEC (104).

In macrophages in NEC, there is an increase in Smad7 expression, particularly in areas with severe intestinal tissue damage and high bacterial concentration. The LPS-induced increase in Smad7 expression suppresses the TGF-β pathway activity and upregulates NF-κB activation via an increased expression of IKK-β by directly activating its promoter in macrophages. This further increases Smad7 expression in a feedback loop of inflammatory activation.

Smad7 is a TGF-β1 receptor antagonist and blocks TGF-β1 (Figures 2, 3). Smad 7 is a key negative regulator of TGF-β signaling and mediates the crosstalk between TGF-β and other signaling pathways. Smad7 presents both anti-fibrotic and anti-inflammatory activities, so that its overexpression has a therapeutic potential for treating fibrosis and inflammation.

Surgically-resected bowel with NEC shows an increased expression of Smad7. Smad7 inhibits the autocrine expression of TGF-beta2 in intestinal epithelial cells in baboon NEC (138, 139). In the healthy intestinal mucosa, macrophages undergo inflammatory downregulation under the influence of both TGF-β1 and TGF-β2.

LPS treatment of RAW264.7 cells blocks the TGF-β2-induced Smad 2 phosphorylation. Smad7 knockdown reverses the LPS-mediated suppression of TGF-β signaling in macrophages. Smad7 augments LPS-induced NF-κB activation in macrophages by increasing IKK-β expression in these cells.

## TLR4 Activates NF-κB in NEC

A strong link has been reported between NF-κB and the pathogenesis of NEC (140). Activation of TLR4 by its ligand LPS leads to the activation of NF-κB. TLR4 activates NF-κB signaling in numerous cells, for examples in kidney (141) and in atherosclerotic plaque (142). In NEC, the LPS-TLR4 pathway also activates the NF-κB signaling (36). In a neonatal NEC model, the mRNA expression of both TLR4 and NF-κB is significantly increased (143). In isolated IECs from the small intestine, TLR4 activates NF-κB in response to LPS.

## Crosstalk Between TLR4 Signaling and PPARγ in NEC

Premature rat pups delivered by abdominal incision on day 20 of gestation (day 21 is considered as full term) were given a single administration of formula milk via an orogastric catheter (144). This NEC model mimics the pathophysiological conditions that triggers the onset of NEC. The incidence of NEC increases with the volume of formula milk. Expression of IκB-α/β and PPARγ mRNA increases in the inflamed intestine. Activation of NFκ B induces the synthesis of inflammation-related proteins. These results could reflect a negative feedback mechanism in response to intestinal inflammation. PPARγ plays an inhibitory role in the inflammatory responses mediated by NFκB (145–148). In NEC, increased PPARγ expression helps inhibit the intestinal inflammation elicited by NF-κB.

In an adult mouse model of NEC (in intestine, by using an ischemia-reperfusion (I/R) model of NEC), activation of PPARγ induces a protective effect on the small bowel during I/R-induced gut injury (149). The PPARγ expression in both jejunum and ileum is significantly increased at 30 min after I/R injury, an increase that returns to baseline after 3 h. NF-κB activity increases during I/R-induced intestinal injury with attenuated response in 15d-PGJ2-pretreated jejunum. PPARγ agonist pretreatment with 15d- PGJ2 is protective for the small bowel during I/R-induced NEC. Intestinal injury is decreased with early activation of PPARγ by its ligand, 15d-PGJ2. This helps attenuate the NF-κB response. Activation of PPARγ with the consequent inhibition of NF-κB expression, could represent a beneficial therapy in premature infants with NEC.

## Genetic Predisposition to NEC in Premature Infants

In premature infants, a genetic predisposition to NEC has been observed (7–9). Genetic studies of NEC concern several candidate genes or factors such as TLR, single immunoglobulin and toll-interleukin 1 receptor (SIGIRR), nucleotide binding oligomerization domain containing protein 2 (NOD2), autophagy-related 16-Like 1 (ATG16L1), mannose binding lectin (MBL), platelet activating factor (PAF), nuclear factor-kappa B, pro-inflammatory cytokines, fucosyltransferase 2 (FUT2), vascular endothelial growth factor (VEGF), arginine and nitric oxide, heparin-binding epidermal growth factor-like growth factor (HB-EGF) (9). Infants of very low birth weight (VLBW) carrying NOD2 loss-of-function mutations present an increased risk of severe gastrointestinal complications, such as NEC. These infants may require surgery and could benefit from NOD2 genotyping with supplementation by means of probiotics (8). In premature infants, inherited abnormalities in the TLR regulation pathway can contribute to NEC susceptibility. A stop mutation (p.Y168X) associated with NEC and a missense variant (p.S80Y) have been reported in SIGIRR, a gene that inhibits the intestinal TLR signaling. SIGIRR inhibits inflammation induced by lipopolysaccharide, a component of Gram-negative bacteria implied in NEC (7).

Possible postnatal mechanisms can suppress abnormal TLR4 activation after birth. Thus, the intestinal mucosa undergoes a strong transition from a sterile protected site toward a permanent colonized surface. In neonates, both protection from bacteria-induced epithelial damage and intestinal epithelial innate immune tolerance involve the microRNA-146a-mediated translational repression and proteolytic degradation of the TLR signaling molecule, interleukin 1 receptor associated kinase 1 (150). The flagellin-dependent IL-8 response of an immature human enterocyte cell line to a bacterial infection is higher than that of a mature enterocyte cell line. The immature enterocytes express a low level of I_κ_B genes. This may favor the pathogenesis of NEC (29).

## NEC Protection Obtained By TLR4 Inhibition in the Intestinal Epithelium

### Innate Immune Response and Protective Effects on NEC

Several human and animal studies suggest that an abnormal activation of the intestinal immune system contributes to the appearance of NEC. In premature infants, inherited defects in the regulation of innate immune processes probably contribute to NEC susceptibility. In these infants, NEC is partly the consequence of an excessive inflammatory response to an initial bacterial colonization due to the immature expression of the gene innate immune response. Probiotics prevent NEC by modulating enterocyte genes that regulate the immune-mediated inflammation (33). Probiotic conditioned media favors maturation of the gene innate immune response, partly explaining their protective effects in NEC (33). The immune receptor nucleotide-binding-oligomerization domain-2 (NOD2) regulates the immune system. NOD2 activation inhibits TLR4 in enterocytes and reverses the effects of TLR4 on intestinal mucosal injury (74). Single-immunoglobulin interleukin-1 receptor-related molecule (SIGIRR) is a transmembrane protein. In intestinal epithelial cells, SIGIRR inhibits inflammation induced by lipopolysaccharide. SIGIRR is a negative regulator of the TLR4 pathway in the developing intestine. Its insufficiency leads to an intestinal TLR hyper-responsiveness and to severe experimental NEC in mice (79). Moreover, it has been observed that VLBW infants having ≥2 NOD2 genetic risk factors of inflammatory intestine disease have an increased risk of NEC (8).

### Beneficial Effects of Breast Milk

Breast milk inhibits NF-κB activation in IECs and thereby plays a protective role against NEC (32, 151). In Ly6c^+^ monocytes, NF-κB inactivation attenuates NEC (152).

### Role of Epidermal Growth Factor (EGF) Signaling

In the intestinal epithelium, breast milk protects against the development of NEC by inhibiting TLR4 through activation of the epidermal growth factor receptor (EGFR) (126). In IEC-6 enterocytes, breast milk protects against NEC by attenuating TLR4 pathway activity through activation of EGF/EGFR signaling and the phosphorylation of GSK-3β (pGSK-3β). This does not occur in mice lacking EGFR. Selective removal of EGF from breast milk reduces its protective properties against NEC. EGF is abundant in breast milk and amniotic fluid and appears to be important for intestinal development (153–157). In the neonatal intestinal epithelium, the amniotic fluid inhibits the TLR4 pathway via EGFR signaling (75). Cetuximab, an EGFR inhibitor, prevents the protection of breast milk via the TLR4 pathway. Breast milk attenuates the TLR4-mediated NF-κB activation by inhibiting GSK-3β *in vitro* (i.e., by phosphorylating GSK-3β) (158–160). In wild-type IEC-6 cells, pre-treatment with either EGF or breast milk, prior to LPS administration, significantly increases phosphorylation of GSK-3β. In IEC-6 cells, lithium chloride increases the phosphorylation of GSK-3β and decreases the expression of TLR4-mediated IL-1β and IL-6. Breast milk reverses the effects of TLR4 on enterocyte apoptosis and proliferation via EGFR and favors phosphorylation of GSK-3β. Breast milk does not protect against NEC-mediated enterocyte apoptosis nor enhances enterocyte proliferation in EGFR^**Δ**IEC^ mice (126). This shows that breast milk activation of EGFR is required to obtain these protective effects.

GSK-3β/β-catenin signaling plays a key role in determining the enterocyte proliferation that occurs in response to EGFR activation (161).

Inactivation of GSK-3β by phosphorylation at serine 9, negatively affects the NF-κB activation (162–164), thus decreasing NF-κB-dependent pro-inflammatory cytokine production (163). Inactivation of GSK-3β leads to the stabilization of β-catenin, a critical factor responsible for intestinal growth and proliferation (161, 165). Formula-feeding in mice induces an increase in non-phosphorylated GSK-3β protein and a decrease in β-catenin in the ileum of NEC newborns.

### Beneficial Effects of PPARγ Agonists in NEC

In a neonatal preterm rat model, the PPARγ agonist pioglitazone (PIO) reduces the development of NEC (166). PIO in a preterm rat model study has shown a decrease in incidence and severity of NEC. In the ileal tract of treated mice, this is associated with an increase in the anti-inflammatory IL-4 and a decrease of the pro-inflammatory IL-12 and INF-γ levels. In an adult mouse model of NEC (using an intestine ischemia-reperfusion (I/R) model of NEC), activation of PPARγ induces a protective effect on the small bowel during I/R-induced gut injury (149). Although PPARγ appears upregulated in NEC (144), PPARγ agonists may help decrease the major inflammatory processes observed in NEC.

### Beneficial Effects of Wnt/β-Catenin Agonists in NEC

Administration of Wnt7b results in the maintenance of intestinal epithelial homeostasis and the avoidance of NEC intestinal injury in mice (24). Intestinal epithelial proliferation is reduced in NEC, but is rescued by Wnt7b administration. Wnt7b reduces the mortality and severity of NEC by increasing intestinal regeneration. Organoids derived from NEC damaged intestine are rescued by Wnt7b supplementation. TLR4 inhibits the canonical Wnt/β-catenin pathway, decreases activation of the Wnt receptor LRP6, and blocks the protective effect of the Wnt3a ligand (123).

### Lithium Chloride

PI3K-Akt phosphorylates GSK-3β and upregulates β-catenin activity.

The TLR4 pathway blocks the PI3K-Akt phosphorylation that allows GSK-3β to remain active (unphosphorylated) and to phosphorylate β-catenin, leading to its destruction into the proteasome. Lithium chloride phosphorylates GSK-3β. Inhibition of GSK-3β with lithium chloride prevents the negative effects of LPS on β-catenin and restores the proliferation of IEC-6 cells (117).

### TGF-β2

TGF-β2 is sequestered in preterm human milk by chondroitin sulfate proteoglycans (139). Enteral supplementation with recombinant TGF-β2 protects mice from experimental NEC-like injury. The TGF-β1 colostrum level is inversely correlated with birth weight and gestational age. The TGF-β2 level is higher than TGF-β1 in the colostrum of maternal breast milk (167). In growth-restricted infants, the decrease in TGF-β2 plays a significant role in feeding intolerance.

## Conclusion

This review of NEC sheds light on the complexity of the pathophysiology of the disease. In particular, the downregulation of the canonical Wnt/β-catenin pathway induced by activation of the LPS-TLR4 systems makes it possible to understand why canonical Wnt agonists attenuate the severity of the disease in experimental NEC models. Some PPARγ agonists can also minimize the deleterious effects of this disease by downregulating NF-κB signaling. This provides us with the hope that current research will lead to the development of new therapeutic avenues to treat this particularly serious disease.

## Author Contributions

All authors listed have made a substantial, direct and intellectual contribution to the work, and approved it for publication.

## Conflict of Interest

The authors declare that the research was conducted in the absence of any commercial or financial relationships that could be construed as a potential conflict of interest.

## Publisher's Note

All claims expressed in this article are solely those of the authors and do not necessarily represent those of their affiliated organizations, or those of the publisher, the editors and the reviewers. Any product that may be evaluated in this article, or claim that may be made by its manufacturer, is not guaranteed or endorsed by the publisher.
